# Learning more from the inter-rater reliability of interstitial fibrosis assessment beyond just a statistic

**DOI:** 10.1038/s41598-023-40221-6

**Published:** 2023-08-15

**Authors:** Peir-In Liang, Wei-Chou Lin, Mei-Chin Wen, Shun-Chen Huang, Pei-Wei Fang, Hao-Wen Chuang, Yi-Jia Lin, Hui-Ping Chien, Huan-Da Chen, Tai-Di Chen

**Affiliations:** 1 Department of Pathology, Kaohsiung Medical University Hospital, Kaohsiung Medical University, Kaohsiung, Taiwan; 2 Department of Pathology, National Taiwan University Hospital, Taipei, Taiwan; 3 Department of Pathology, China Medical University Hsinchu Hospital, Hsinchu, Taiwan; 4 Department of Anatomic Pathology, Chang Gung Memorial Hospital Kaohsiung Branch, Kaohsiung, Taiwan; 5 Department of Pathology, Fu Jen Catholic University Hospital, Fu Jen Catholic University, New Taipei City, Taiwan; 6 Department of Pathology and Laboratory Medicine, Kaohsiung Veterans General Hospital, Kaohsiung, Taiwan; 7 Department of Pathology, Tri-service General Hospital, National Defense Medical Center, Taipei, Taiwan; 8 Department of Pathology and Laboratory Medicine, Shin Kong Wu Ho-Su Memorial Hospital, Taipei, Taiwan; 9 Department of Anatomic Pathology, Chang Gung Memorial Hospital Linkou Main Branch, Taoyuan, Taiwan

**Keywords:** Nephritis, Renal fibrosis

## Abstract

Interstitial fibrosis assessment by renal pathologists lacks good agreement, and we aimed to investigate its hidden properties and infer possible clinical impact. Fifty kidney biopsies were assessed by 9 renal pathologists and evaluated by intraclass correlation coefficients (ICCs) and kappa statistics. Probabilities of pathologists’ assessments that would deviate far from true values were derived from quadratic regression and multilayer perceptron nonlinear regression. Likely causes of variation in interstitial fibrosis assessment were investigated. Possible misclassification rates were inferred on reported large cohorts. We found inter-rater reliabilities ranged from poor to good (ICCs 0.48 to 0.90), and pathologists’ assessments had the worst agreements when the extent of interstitial fibrosis was moderate. 33.5% of pathologists’ assessments were expected to deviate far from the true values. Variation in interstitial fibrosis assessment was found to be correlated with variation in interstitial inflammation assessment (r^2^ = 32.1%). Taking IgA nephropathy as an example, the Oxford T scores for interstitial fibrosis were expected to be misclassified in 21.9% of patients. This study demonstrated the complexity of the inter-rater reliability of interstitial fibrosis assessment, and our proposed approaches discovered previously unknown properties in pathologists’ practice and inferred a possible clinical impact on patients.

## Introduction

The practice of diagnostic kidney biopsy has adopted numerous quantitative classification systems or grading schemes to provide prognostic or predictive information. Reproducibility, evaluated by inter-rater reliability and intra-rater reliability, is generally considered vastly important when constructing and proposing such a classification or grading scheme. Variations of the findings in subsequent validation studies on proposed schemes might partly reflex differences in the ways targeting variables are assessed, and reproducibility is likely to be even lower in daily practice^1,2^.

Interstitial fibrosis observed in kidney biopsy is one of the most important pathological prognostic variables for renal outcomes in patients with chronic kidney disease^3^. Interstitial fibrosis assessment is routinely implemented in various reporting guidelines^4–8^. Given its importance, good agreement is essential. However, the inter-rater reliability of interstitial fibrosis assessment has only been investigated, mostly as a minor appendant part, in few publications^1,2,9–17^. The reported values are quite variable, with reliability coefficients ranging from 0.10 to 0.98 for intraclass correlation coefficients (ICCs) and from 0.25 to 0.53 for kappa statistics. In addition, interstitial fibrosis assessment has been shown inconsistent even among expert renal pathologists^9,16,17^.

Because the study populations, study designs, and results of published literature are heterogeneous, how well or poorly renal pathologists perform on interstitial fibrosis assessment is not well understood, reasons for good or poor inter-rater reliability have not been investigated, and to what extent the variation in assessment may impact clinical practice is unknown. To address these issues, in this study we proposed novel approaches to interpret the data beyond just reporting a single reliability coefficient.

## Methods

### Datasets

Kidney allograft biopsies between 2018 and 2020 obtained at Linkou Chang Gung Memorial Hospital were used. Fifty cases representing the full spectrum of interstitial fibrosis were selected from the archive for the assessment by one senior renal pathologist (T.C.), who has over 10 years of experience signing out 3000+ kidney biopsies independently. Slides were scanned and converted to whole slide imaging (WSI) with a NanoZoomer S360 Digital slide scanner C13220-01 at 40× mode with scanning resolution of 0.23 μm/pixel. The study was approved by the Chang Gung Medical Foundation Institutional Review Board (IRB No.: 202200101B0). The study adhered to the Declaration of Helsinki.

### Interstitial fibrosis assessment by pathologists

Four senior and five junior renal pathologists from 9 different hospitals in Taiwan participated in the study. All pathologists were provided with WSIs of each case and conducted interstitial fibrosis assessment according to their usual practice. The assessments were reported as interstitial fibrosis percentages. For each case, the average of all 9 pathologists’ assessments was taken as the true value of interstitial fibrosis. To obtain ci scores of the Banff scheme for kidney allograft biopsy and T scores of the Oxford classification for IgA nephropathy, interstitial fibrosis percentages of each case were translated to scorings^5,18^. To investigate possible causes for the variation in interstitial fibrosis assessment, cortical area percentage and total inflammation area percentage were also assessed by all participating pathologists on the same 50 cases.

### Intuitive representations of estimated deviation rates and misclassification rates

The true value of the interstitial fibrosis percentage (x) ± an arbitrary margin of 10 was set as the acceptable range for interstitial fibrosis percentage assessments. Assessments falling outside of (x ± 10) were considered significant deviations. The “margin” can be of any value depending on how narrow we consider the acceptable range should be. We chose 10% because it is a common practice for pathologists to assess the interstitial fibrosis percentage to the nearest 10%. For each case, the probability of assessments within the defined acceptable range from 9 pathologists was plotted along the Y-axis, with the X-axis representing the true value of interstitial fibrosis. A univariate quadratic function was fitted to the scatterplot to obtain the regression formula ŷ = 105.4040 − 1.9802x + 0.0180x^2^. The estimated deviation rate for a given true value of interstitial fibrosis percentage x was calculated by 1 – ${\hat{\text{y}}}$. The overall probability of a random pathologist making an acceptable assessment for a random case was the integral between x = 0 and x = 100 under the regression curve, and the overall deviation rate was the integral above the regression curve.

For ordinal data such as Banff ci scores and Oxford T scores, we defined that the assessment was correct when the score reported by the pathologist was the same as the score translated from the true value of interstitial fibrosis percentage x. Other scores were considered misclassified. For each case, the cumulative probability that a score would take a value less than or equal to the true score was plotted along the Y-axis, with the X-axis representing the true value of interstitial fibrosis. Nonlinear regression by multilayer perceptron (Supplementary Method) was applied to obtain regression functions of each score’s boundaries. The estimated probability of reporting a particular score was the area between the score’s X-axis and regression curves inferred by the multilayer perceptron divided by the total area between the score’s X-axis boundaries. The misclassification rate for a given true Banff ci or Oxford T score was calculated by 1 − (the probability of reporting a correct score).

### Identifying possible causes for the variation in interstitial fibrosis assessment

Cases with exceptionally good agreement and cases difficult for pathologists to concur within the range of moderate interstitial fibrosis were identified. An unpaired t test was applied to evaluate whether the variances in interstitial fibrosis assessment of these representative cases significantly differed. These cases were reviewed, and the feedback from pathologists indicated that the difference in variances may come from (1) difficulty delineating the cortical area in the biopsy and (2) the presence of marked inflammation obscuring the underlying interstitium. Unpaired t tests were applied on these representative cases to validate the above observations, and Pearson correlation coefficient calculations were applied on all 50 cases for correlations of the interstitial fibrosis assessment variance with (1) the average cortical area percentage, (2) the average total inflammation area percentage, and (3) the assessment variance in total inflammation area percentage.

### Inferring clinical impact on published datasets

Publications regarding the Oxford classification for IgA nephropathy were searched on PubMed^®^ and reviewed. To obtain a good representation of the population distribution of interstitial fibrosis, only publications with 500 cases or more were included in the evaluation^19–23^. Case numbers belonging to each Oxford T score were retrieved and multiplied by misclassification rates estimated as mentioned above.

### Statistics

Inter-rater reliabilities for interstitial fibrosis percentage were evaluated by ICC (2,1) for either pairwise agreement between any two raters or the overall agreement among all 9 raters. Inter-rater reliabilities for Banff ci or Oxford T scores were evaluated by linearly weighted Cohen’s kappa for pairwise agreement between any two raters. The relationships between the variance in interstitial fibrosis assessment and average cortical area percentage, average total inflammation area percentage, and assessment variance in total inflammation area percentage were evaluated as described in the above section. The proportion of variation in interstitial fibrosis assessment that is explained by identified variables was measured by R-squared in a multiple linear regression model. All analyses were conducted using SPSS 26 (IBM SPSS Inc, Chicago, Illinois).

### Ethical approval

The studies involving human participants were reviewed and approved by Chang Gung Medical Foundation Institutional Review Board. Written informed consent was waived by the Chang Gung Medical Foundation Institutional Review Board.

## Results

### Pairwise inter-rater reliabilities of interstitial fibrosis assessment had a wide range

**Figure 1:**
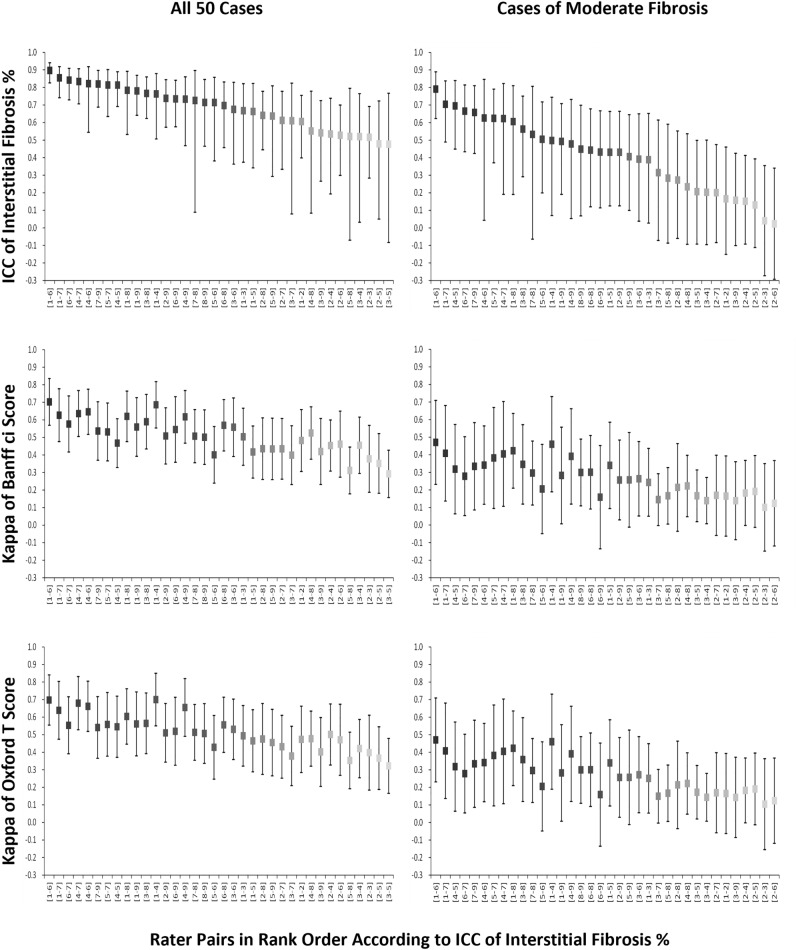
Pairwise inter-rater reliabilities of the interstitial fibrosis assessment of the 9 pathologists placed in rank order by ICCs. Left side: all 50 cases; right side: only cases of moderate (25–75%) interstitial fibrosis. Boxes with the same colour in each graph indicate the same pair of pathologists.

Figure 1 shows the pairwise inter-rater reliabilities of the interstitial fibrosis assessment of the 9 pathologists placed in rank order by ICC. The pairwise ICCs of interstitial fibrosis percentage ranged from 0.48 (poor) to 0.90 (good). The overall ICC of all 9 pathologists was 0.68 with a 95% confidence interval (CI) of 0.57 (moderate) to 0.79 (good). For the 4-tier interstitial fibrosis score translated from the interstitial fibrosis percentage according to the Banff scheme (ci0, ci1, ci2, and c3), the pairwise weighted kappa coefficients ranged from 0.29 (fair) to 0.70 (substantial). For the 3-tier score translated according to the Oxford classification for IgA nephropathy (T0, T1, and T2), the pairwise weighted kappa coefficients ranged from 0.32 (fair) to 0.70 (substantial). Senior pathologists did not perform better than junior pathologists (ICCs: 0.67 vs. 0.66). A summary of ICCs and kappa statistics in each experimental condition is shown in Table 1. Please refer to Supplementary Table 1 for interstitial fibrosis assessment raw results. Given the strong correlation between interstitial fibrosis and tubular atrophy in the original Oxford classification report, tubular atrophy might serve as a surrogate for interstitial fibrosis if it has better inter-rater reliability. However, it was not the case (Supplementary Table 2).

**Table 1:** Summary of ICCs and kappa statistics in each experimental condition.

	All raters (n = 9)	Senior raters (n = 4)	Junior raters (n = 5)
ICC			
On all cases (interstitial fibrosis 0–100%)	0.68 (0.57–0.79)	0.67 (0.49–0.79)	0.66 (0.50–0.78)
On cases with moderate fibrosis (interstitial fibrosis 25–75%)	0.40 (0.26–0.56)	0.35 (0.15–0.56)	0.36 (0.20–0.54)
Pairwise weighted kappa (for Banff ci score)			
Average on all cases (interstitial fibrosis 0–100%)	0.50 ± 0.10	0.54 ± 0.10	0.46 ± 0.09
Average on cases with moderate fibrosis (interstitial fibrosis 25–75%)	0.27 ± 0.10	0.28 ± 0.13	0.23 ± 0.08
Pairwise weighted kappa (for Oxford T score)			
Average on all cases (interstitial fibrosis 0–100%)	0.51 ± 0.10	0.54 ± 0.12	0.47 ± 0.08
Average on cases with moderate fibrosis (interstitial fibrosis 25–75%)	0.27 ± 0.10	0.28 ± 0.13	0.23 ± 0.08

All comparisons between senior raters and junior raters were not statistically significant. ICCs (intraclass correlation coefficients) showing in estimated value (95% confidence interval). Kappa statistics showing in mean ± standard deviation.

### Worse inter-rater reliabilities for cases of moderate interstitial fibrosis

**Figure 2:**
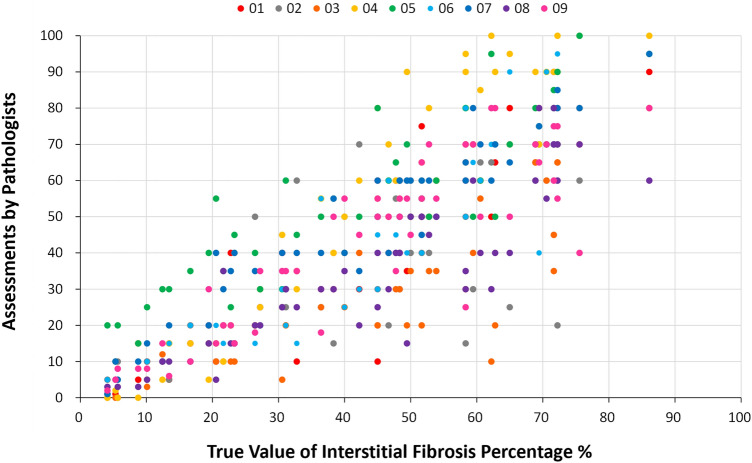
Scatterplot of interstitial fibrosis assessments from the 9 pathologists on 50 cases. The X-axis is the true value of interstitial fibrosis percentage determined by averaging 9 assessments of each single case. Assessments by senior pathologists are shown in warm colours, and those by junior pathologists are shown in cool colours. Note that assessments converged at both ends and diverged in the middle of the measurement range.

Figure 2 shows the scatterplot of assessments from the 9 pathologists on all 50 cases in rank order according to the true value of interstitial fibrosis percentage of each case. It is visually evident that variances in assessments differed throughout the measurement range, as the distributions of assessments converged at both ends but diverged in the middle where the extent of interstitial fibrosis is moderate. A lower ICC (0.40; 95% CI 0.26 to 0.56), lower average weighted pairwise kappa for Banff ci scores (0.27 ± 0.10), and lower average weighted pairwise kappa value for Oxford T scores (0.27 ± 0.10) for cases of moderate (25–75%) interstitial fibrosis confirmed the observation (Table 1). In agreement with assessments on the full measurement range, senior pathologists did not perform better than junior pathologists (ICC 0.35 vs. 0.36).

### Intuitive representations of estimated deviation rates

**Figure 3:**
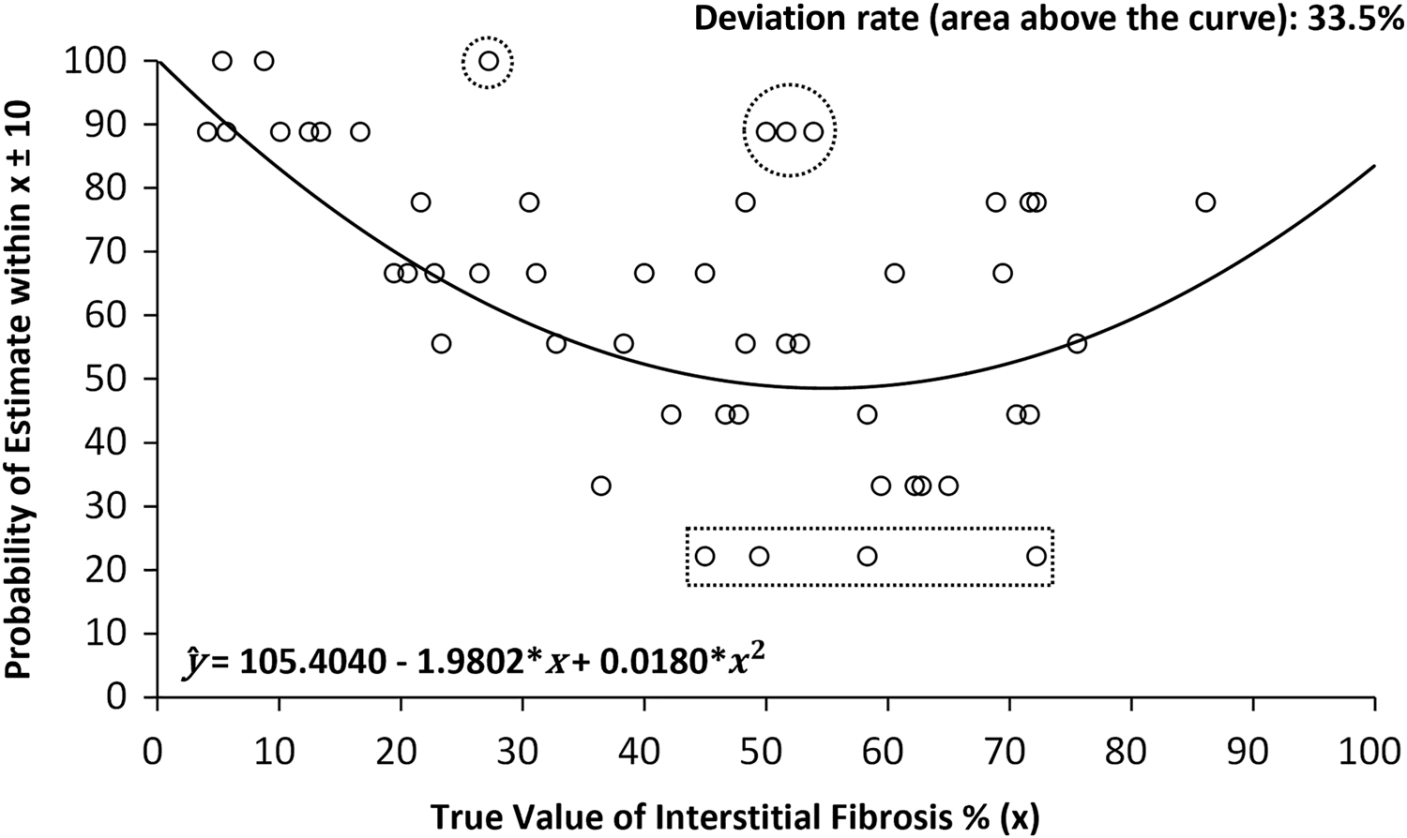
Curve chart showing the probability of making acceptable assessments (within true value x ± 10). Dots in circles are cases of moderate interstitial fibrosis with exceptionally good concordance, and dots in the rectangle are cases with very poor concordance.

Figure 3 shows the estimated probability (${\hat{\text{y}}}$) of the assessment made by a random pathologist would fall inside the acceptable range (x ± 10) when a true value of interstitial fibrosis percentage x is given. For example, if the true value of interstitial fibrosis percentage is 30, the probability of the assessment made by a random pathologist would fall inside the acceptable range of 20–40 is 62.2%. If the true value of interstitial fibrosis percentage is 50, the probability of the assessment within the acceptable range of 40–60 is 51.4%. Across the whole measurement range, the overall probability of a pathologist would make an acceptable assessment is 66.5% (thus, the deviation rate is 33.5%), and it is visually evident that pathologists did worse when the extent of interstitial fibrosis was in the middle of the measurement range. Supplementary Fig. 1 provides examples for stricter (x ± 5) and looser (x ± 15) acceptable ranges, which shows that the deviation rate is even higher with a stricter range.

### Intuitive representations of estimated misclassification rates

**Figure 4:**
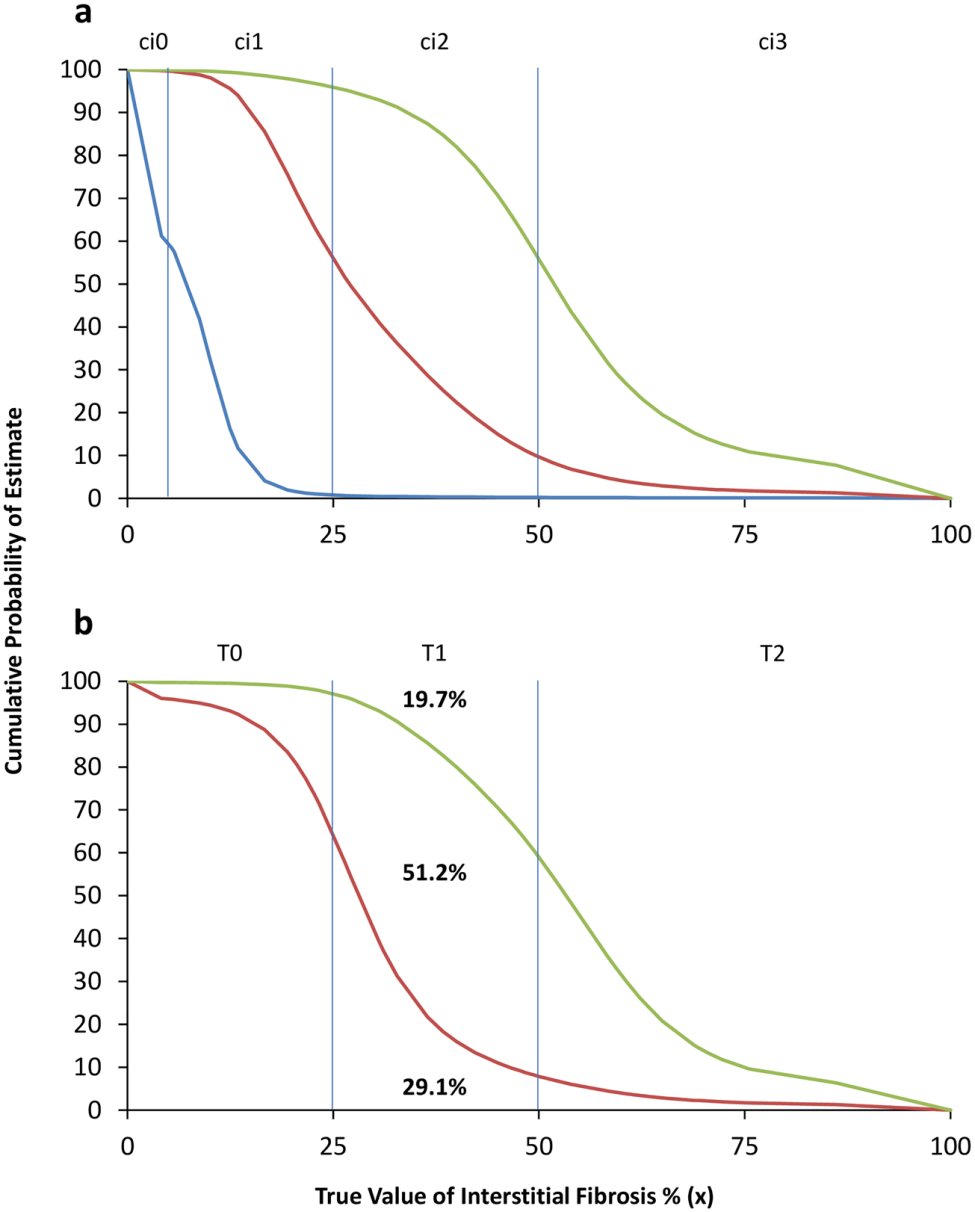
Curve chart showing the probabilities of making a correction classification or a misclassification when Banff ci scores (**a**) and Oxford T scores (**b**) were applied. For each probability value, please refer to Table 2. Taking the Oxford T1 score (x = 25–50) as an example, the area under the red curve represents the probability of undergrading (29.1%), and the area above the green curve represents the probability of overgrading (19.7%). The area between the red curve and the green curve is the probability (51.2%) that a random pathologist would make a correct classification. Curves represent the cumulative probability that the score assigned by a random pathologists would take a value less than or equal to the true score; blue: ci0; red: ci1 for Banff and T0 for Oxford; green: ci2 for Banff and T1 for Oxford.

Figure 4 visualizes the estimated probabilities of correct classification and misclassification of Banff ci scores and Oxford T scores reported by a random pathologist, and Table 2 summarizes the results. Taking the Oxford T score as an example, the probability of a pathologist would make a correct classification was 84.6% for T0 score, 51.2% for T1 score, and 81.4% for T2 score. For the T1 score, the probability of underscoring (to T0) was 29.1% and of overscoring (to T2) was 19.7%. For the T2 score, the probability of underscoring was the combination of misclassification to T0 and T1 (2.4% + 16.2% = 18.6%).

**Table 2:** Estimated misclassification rates of Banff ci scores and Oxford T scores.

		True ci score of Banff scheme			
		ci 0 (≤ 5%)	ci 1 (6–25%)	ci2 (26–50%)	ci3 (> 50%)
Score by a random rater	ci 3	0.1%	1.4%	17.6%	**82.6%**
	ci 2	0.1%	13.4%	**53.8%**	14.6%
	ci 1	36.9%	**67.6%**	28.2%	2.6%
	ci 0	**62.9%**	17.6%	0.4%	0.2%

		True T score of Oxford classification			
		T0 (≤ 25%)	T1 (26–50%)	T2 (> 50%)	
Score by a random rater	T2	1.8%	19.7%	**81.4%**	
	T1	13.6%	**51.2%**	16.2%	
	T0	**84.6%**	29.1%	2.4%	

Percentages in bold are correct classifications.

### Identifying possible causes for the variation in interstitial fibrosis assessment

Although the inter-rater reliability of cases with moderate interstitial fibrosis was poor in general, carefully examining Fig. 3 allowed us to identify cases with good agreement and cases in which it was difficult for pathologists to concur with each other. A *t*-test confirmed that the variances in the interstitial fibrosis assessment of these two groups were significantly different (59.2 vs 650.9; *P* < 0.0001). *t*-tests also confirmed that both the average total inflammation area percentage (19.1 vs 46.2) and the variance in total interstitial inflammation area percentage (144.1 vs 598.7) were significantly higher in difficult cases (*P* = 0.012 and *P* = 0.039). Verifying all 50 cases, significant correlations were found between the variance in interstitial fibrosis assessment and both variables (r = 0.47 and r = 0.56, respectively). These two factors accounted for 32.1% of the total variance in interstitial fibrosis assessment on multiple linear regression (Table 3).

**Table 3:** Possible causes for the variation in interstitial fibrosis assessment.

Parameters	Crude *B* (95% CI)	*P*	*r*^2^	Parameters	Adjusted *B* (95% CI)	*P*	*r*^2^
Intercept	41.2 (− 65.2, 147.7)	0.440	22.4%	Intercept	53.2 (− 47.9, 154.3)	0.295	32.1%
ti average	5.8 (2.6, 8.9)	**0.001**		ti average	1.4 (− 3.0, 5.9)	0.522	
				ti variance	0.3 (0.1, 0.6)	**0.013**	
Intercept	74.3 (− 2.2, 150.7)	0.057	31.5%				
ti variance	0.4 (0.2, 0.6)	** < 0.001**					

Values in bold are statistically significant. Univariate analysis on the left side and multivariate analysis on the right side. ti, total interstitial inflammation.

### Inferring clinical impact on published datasets

The T score of the Oxford classification has repeatedly been shown to be an independent prognostic variable of patients with IgA nephropathy. Case numbers of different T scores in IgA nephropathy were retrieved from publications of large cohorts (n ≥ 500)^19–23^. Taking the T scores reported in publications as gold standards, the possible misclassification rates of the Oxford T score in real-world practice were inferred (Table 4). Downgrading rates were estimated to range from 4.4 to 8.0%, and upgrading rates were estimated to range from 14.3 to 15.5%. Overall, 21.9% of patients with IgA nephropathy were possibly subjected to misclassification of the Oxford T score and might have had incorrect renal outcome prognostication and/or inappropriate clinical management. We conducted the same analysis on two large kidney allograft biopsy cohorts and found similar results (Supplementary Table 3).

**Table 4:** Inferred misclassification rates of Oxford T scores in reported large cohorts.

			True score				Misclassification	
			T0	T1	T2		Number	%
2021 Hwang et al.^23^			378	120	47			
(545)	*Inferred score*	*T0*	**320**	35	1	Downgrade	44	8.0
		*T1*	51	**61**	8	Upgrade	82	15.0
		*T2*	7	24	**38**	Total	126	23.0
2020 Moriyama et al.^22^			631	189	51			
(871)	*Inferred score*	*T0*	**534**	55	1	Downgrade	64	7.4
		*T1*	86	**97**	8	Upgrade	134	15.4
		*T2*	11	37	**42**	Total	199	22.8
2017 Hass et al.^21^			2322	619	155			
(3096)	*inferred score*	*T0*	**1964**	180	4	Downgrade	209	6.7
		*T1*	316	**317**	25	Upgrade	480	15.5
		*T2*	42	122	**126**	Total	688	22.2
2014 Park et al.^20^			411	53	36			
(500)	*Inferred score*	*T0*	**348**	15	1	Downgrade	22	4.4
		*T1*	56	**27**	6	Upgrade	74	14.7
		*T2*	7	10	**29**	Total	96	19.2
2013 Tanaka et al.^19^			551	98	49			
(698)	*Inferred score*	*T0*	**466**	29	1	Downgrade	38	5.4
		*T1*	75	**50**	8	Upgrade	104	14.9
		*T2*	10	19	**40**	Total	142	20.3
			496	123	83			
(702)	*Inferred score*	*T0*	**420**	36	2	Downgrade	51	7.3
		*T1*	67	**63**	13	Upgrade	101	14.3
		*T2*	9	24	**68**	Total	152	21.6
All			4789	1202	421			
(6412)	*Inferred score*	*T0*	**4051**	350	10	Downgrade	428	6.7
		*T1*	651	**615**	68	Upgrade	974	15.2
		*T2*	86	237	**343**	Total	1402	21.9

Numbers in parentheses are the total number of patients in publications. True T scores reported in publications are underlined. Inferred scores in real-world practice are shown in italics. Case numbers in bold are correct classifications. The estimated misclassified case numbers and rates are shown on the right-hand side.

## Discussion

Interstitial fibrosis is one of the most relevant pathological variables associated with renal outcome. Given its critical role, it is surprising that literature focusing on inter-rater reliability of interstitial fibrosis assessment is scarce. In this study, we confirmed previous observations that pathologists’ assessments of interstitial fibrosis are variable. Pairwise agreements range from nearly optimal to simply not acceptable. We found no evidence that senior pathologists perform better than their junior colleagues. We showed that inter-rater reliability is not a constant value along the measurement range. Moreover, several points should be further expounded upon, as discussed below.

Previous studies frequently utilized ICCs or kappa statistics to investigate inter-rater reliability. Some studies calculated ICCs from ordinal data to draw their conclusions, which is inappropriate^12,15,17^. Ordinal data as a kind of categorical rating are not compatible with the assumptions of ICC, and weighted chance-corrected agreement coefficients such as weighted kappa or Gwet’s AC_1_ should be applied in this setting^24^.

The interstitial fibrosis percentage is a kind of ratio rating; therefore, investigating by ICC is appropriate. However, there are several disadvantages. First, ICCs cannot be compared between samples drawn from different populations. ICC is a ratio of subject variance as the numerator and total variance as the denominator. Since study samples from different populations likely have different subject variances, ICCs derived from different studies cannot be compared. This could be one of the reasons why ICCs of interstitial fibrosis assessment reported in publications vary widely. Therefore, a general understanding for the inter-rater reliability of interstitial fibrosis assessment cannot be drawn from existing literature.

Another problem with ICC is that it cannot capture the fact that inter-rater reliability changes along the measurement range, which is evident on a scatterplot. As a summary statistic, all differences in variances of reliability along the whole measurement range are concluded to a single value when calculating ICCs. This property conceals the variation in inter-rater reliability. Ignoring this fact will result in misleading interpretations when the underlying population distribution of the target variable is taken into consideration.

Most importantly, ICCs and kappa statistics are obscured to practising nephrologists and pathologists. Although benchmarks have been proposed^25,26^, interpretations still highly depend on understanding study designs, domain expertise, and statistical knowledge.

In this study, we demonstrated novel, complementary approaches to overcome the abovementioned problems. Our approaches start with defining what counts as a good assessment and then examining pathologists’ performance at different extents or within different categories of interstitial fibrosis. With our approaches, even without statistical knowledge, one can easily observe that the inter-rater reliability is good when the extent of interstitial fibrosis is limited, gradually worsens with the increase in fibrosis up to 50–60%, and bounces back again thereafter. The regression formulas, not depending on a particular sample distribution such as ICC, can be applied to other cohorts to infer the probabilities of assessment deviation rates and misclassification rates. An additional benefit of the proposed approaches is that cases with extraordinarily better or worse assessment concordance can be easily identified and analysed for possible causes of poor agreement. A pathologists’ intuition on the assessment of interstitial fibrosis is that the presence of interstitial inflammation can make precise evaluation impossible, which was confirmed in the current study. Interestingly, by multiple linear regression, we showed that it is not the extent of interstitial inflammation but the variation in how much inflammation in the biopsy assessed by pathologists correlates with the disagreement of interstitial fibrosis assessment. Other factors possibly contributing to inconsistent interpretations, such as experience of the pathologists, fixed tendencies of over- or under-estimation of the pathologists, or failure to accurately delineate the extent of cortical area in biopsies before the interpretation of fibrosis, might deserve exploration in future work.

Our approaches also provide a novel way to investigate the clinical impact of variation in interstitial fibrosis assessment on kidney biopsy cohorts. The importance of the interstitial fibrosis T score of the Oxford classification in patients with IgA nephropathy has been validated in many large studies. If the distribution of the extent of interstitial fibrosis in these studies was considered accurate, then we can infer that in real-world practice, up to 21.9% of IgA nephropathy patients would have their extent of interstitial fibrosis misclassified and received incorrect prognostication or even inappropriate treatment.

The inter-rater reliability assessed based on 50 cases by 9 renal pathologists of different levels of experience from different hospitals should be representative of general practice in the real world^24^. Nonetheless, the current findings would be better validated by future studies with different numbers of participants and cases. Although our approaches provide a clearer view of the properties of the inter-rater reliability of interstitial fibrosis assessment and point out its possible clinical implications, how to ameliorate these identified issues and achieve better agreement requires more investigation. It is intuitive that senior pathologists may have better concordance than those with less experience, but we did not observe this difference. In addition, it has been shown that the reliability of human assessment does not improve even after persistent feedback^11^. In this regard, human assessment might have reached its limit, and recently described machine learning-based methodologies might provide promising mediation^27–29^. Alternatively, one may consider investigating the possibilities of predicting disease progression based on the expression of genes or proteins in the biopsy, bypassing the interpretation of morphology entirely.

In conclusion, in this study, we proposed novel approaches to investigate the complex nature of the inter-rater reliability of interstitial fibrosis assessment on kidney biopsies. The proposed approaches provide complementary, more detailed, and easily interpretable information in addition to the traditional reliability metrics. We found factors correlated with the variation in interstitial fibrosis assessment and showed how to generalize the findings from our dataset to estimate deviation/misclassification rates for inferring clinical impacts. The evaluation of inter-rater reliability is very important not only in medicine but also in all scientific fields involving human interpretation and judgement. Our findings may encourage more understandable and meaningful reporting on inter-rater reliability in a wide array of future studies.

### Supplementary Information

Supplementary Information.

## Data Availability

The datasets generated during and/or analysed during the current study are available from the corresponding author on reasonable request.

## References

[1] Bellur SS (2019). Reproducibility of the Oxford classification of immunoglobulin A nephropathy, impact of biopsy scoring on treatment allocation and clinical relevance of disagreements: Evidence from the VALidation of IGA study cohort. Nephrol. Dial. Transplant..

[2] Working Group of the International IgANN (2009). The Oxford classification of IgA nephropathy: Pathology definitions, correlations, and reproducibility. Kidney Int..

[3] Farris AB (2014). Banff fibrosis study: Multicenter visual assessment and computerized analysis of interstitial fibrosis in kidney biopsies. Am. J. Transplant..

[4] Solez K (1993). International standardization of criteria for the histologic diagnosis of renal allograft rejection: The Banff working classification of kidney transplant pathology. Kidney Int..

[5] Working Group of the International IgANN (2009). The Oxford classification of IgA nephropathy: Rationale, clinicopathological correlations, and classification. Kidney Int..

[6] Tervaert TW (2010). Pathologic classification of diabetic nephropathy. J. Am. Soc. Nephrol..

[7] Sethi S (2016). Mayo clinic/renal pathology society consensus report on pathologic classification, diagnosis, and reporting of GN. J. Am. Soc. Nephrol..

[8] Bajema IM (2018). Revision of the International Society of Nephrology/Renal Pathology Society classification for lupus nephritis: Clarification of definitions, and modified National Institutes of Health activity and chronicity indices. Kidney Int..

[9] Furness PN, Taub N, Convergence of European Renal Transplant Pathology Assessment Procedures, P (2001). International variation in the interpretation of renal transplant biopsies: Report of the CERTPAP Project. Kidney Int..

[10] Gough J (2002). Reproducibility of the Banff schema in reporting protocol biopsies of stable renal allografts. Nephrol. Dial. Transplant..

[11] Furness PN (2003). International variation in histologic grading is large, and persistent feedback does not improve reproducibility. Am. J. Surg. Pathol..

[12] Snoeijs MG (2010). Histological assessment of pre-transplant kidney biopsies is reproducible and representative. Histopathology.

[13] Herzenberg AM (2011). Validation of the Oxford classification of IgA nephropathy. Kidney Int..

[14] Hisano S (2017). Reproducibility for pathological prognostic parameters of the Oxford classification of IgA nephropathy: A Japanese cohort study of the Ministry of Health, Labor and Welfare. Clin. Exp. Nephrol..

[15] Liapis H (2017). Banff Histopathological consensus criteria for preimplantation kidney biopsies. Am. J. Transplant..

[16] Grootscholten C (2008). Interobserver agreement of scoring of histopathological characteristics and classification of lupus nephritis. Nephrol. Dial. Transplant..

[17] Oni L (2017). Inter-observer variability of the histological classification of lupus glomerulonephritis in children. Lupus.

[18] Roufosse C (2018). A 2018 reference guide to the Banff classification of renal allograft pathology. Transplantation.

[19] Tanaka S (2013). Development and validation of a prediction rule using the Oxford classification in IgA nephropathy. Clin. J. Am. Soc. Nephrol..

[20] Park KS (2014). Comparison of the Haas and the Oxford classifications for prediction of renal outcome in patients with IgA nephropathy. Hum. Pathol..

[21] Haas M (2017). A multicenter study of the predictive value of crescents in IgA nephropathy. J. Am. Soc. Nephrol..

[22] Moriyama T (2020). Validation of the revised Oxford classification for IgA nephropathy considering treatment with corticosteroids/immunosuppressors. Sci. Rep..

[23] Hwang D (2021). Validation of an international prediction model including the Oxford classification in Korean patients with IgA nephropathy. Nephrology.

[24] Gwet, K. L. *Handbook of Inter-Rater Reliability—The Definitive Guide to Measuring the Extent of Agreement Among Raters.* 5 edition. *Advanced Analytics, LLC.* Vol. **1**, (2021).

[25] Landis JR, Koch GG (1977). The measurement of observer agreement for categorical data. Biometrics.

[26] Koo TK, Li MY (2016). A guideline of selecting and reporting intraclass correlation coefficients for reliability research. J. Chiropr. Med..

[27] Ginley B (2021). Automated computational detection of interstitial fibrosis, tubular atrophy, and glomerulosclerosis. J. Am. Soc. Nephrol..

[28] Zheng Y (2021). Deep-learning-driven quantification of interstitial fibrosis in digitized kidney biopsies. Am. J. Pathol..

[29] Liu ZY (2022). End-to-end interstitial fibrosis assessment of kidney biopsies with a machine learning-based model. Nephrol. Dial. Transplant..

