# Supplementary material for: Learning more from the inter-rater reliability of interstitial fibrosis assessment beyond just a statistic
Source: Sci Rep. 2023 Aug 15;13:13260. doi: 10.1038/s41598-023-40221-6 (PMC10427633; doi:10.1038/s41598-023-40221-6)

Supplementary material to:

Learning more from the inter-rater reliability of interstitial fibrosis assessment beyond just a statistic

Supplementary Method: Nonlinear Regression by Multilayer Perceptron

Nonlinear regression by multilayer perceptron was applied to each score boundary (5%, 25%, and 50% for Banff ci scores and 25% and 50% for Oxford T scores) to obtain each boundary's regression function. The input of multilayer perceptron were the true values of interstitial fibrosis. The number of hidden layers was set to 1 and both the activation functions for hidden and output layers were set as sigmoid. Seventy percent (n=35) of the cases were used for training and thirty percent (n=15) of the cases were for testing. The response variables were the cumulative percentage (observed probability) at 5%, 25% and 50% for Banff ci scores and at 25% and 50% for Oxford T scores. The estimated probability of reporting a particular score was the area between the score's X-axis and regression curves inferred by the multilayer perceptron divided by the total area between the score's X-axis boundaries. For example, the probability of reporting a correct score of Oxford T1 (interstitial fibrosis = 25% to 50%) was the area between x = 25 and x = 50 along the X-axis and regression curves of y = 25 and y = 50, divided by the total area between x = 25 and x = 50. The misclassification rate for a given true Banff ci or Oxford T score was calculated by 1 - (the probability of reporting a correct score). The analysis was conducted using SPSS 26 (IBM SPSS Inc, Chicago, Illinois).

Supplementary Table 1. Interstitial fibrosis assessment raw results

			Raters								
			Senior				Junior				
Case	Average	SD	01	03	04	09	02	05	06	07	08
01	48	13	60	30	60	35	55	65	45	40	40
02	76	19	70	80	100	40	60	100	80	80	70
03	69	12	70	80	70	65	70	75	40	75	80
04	42	16	30	40	60	45	70	50	30	35	20
05	33	15	10	35	30	35	60	45	15	40	25
06	45	25	10	20	80	55	30	80	30	60	40
07	6	6	0	0	0	8	10	20	5	5	3
08	9	6	5	15	0	8	15	15	8	10	3
09	10	7	10	3	5	8	10	25	10	15	5
10	21	16	10	10	15	15	15	55	20	40	5
11	26	12	20	20	20	18	50	40	15	35	20
12	22	10	20	10	10	20	20	35	15	30	35
13	12	7	10	12	5	15	10	30	10	10	10
14	17	8	15	15	15	10	20	35	20	10	10
15	50	9	50	35	60	45	40	50	60	60	50
16	13	8	5	15	15	6	5	30	15	20	10
17	4	6	1	0	0	2	5	20	5	1	3
18	48	12	60	30	55	55	30	60	55	50	40
19	48	9	60	40	40	50	40	50	55	60	40
20	72	17	90	35	80	60	60	85	75	80	80
21	31	12	40	5	45	35	25	30	30	40	25
22	71	12	70	60	90	70	60	70	90	70	55
23	72	9	70	65	90	75	60	70	70	80	70
24	53	15	50	35	80	70	40	50	45	60	45
25	38	13	30	40	40	50	15	55	30	55	30
26	19	10	20	15	5	30	15	40	15	20	15
27	65	23	80	70	95	50	25	70	90	65	40
28	5	4	1	0	2	5	10	10	5	10	5
29	23	14	15	10	40	15	15	45	15	40	15
30	27	6	25	30	25	35	25	30	35	20	20
31	54	8	60	35	60	55	60	60	55	50	50
32	52	8	50	55	60	65	50	50	40	45	50
33	58	27	70	65	95	25	15	80	80	60	35
34	40	11	40	25	50	55	50	40	25	40	35
35	31	12	30	20	30	35	25	60	20	30	30
36	47	16	40	50	70	50	20	60	60	40	30
37	61	13	60	55	85	50	65	60	60	70	40

38	36	14	30	25	55	18	25	50	55	40	30
39	23	9	40	10	20	20	20	25	20	35	15
40	45	13	60	20	55	50	50	50	45	50	25
41	69	10	65	65	90	70	60	80	60	70	60
42	49	24	35	20	90	55	60	70	40	60	15
43	58	21	60	30	90	70	50	80	50	65	30
44	86	12	90	80	100	80	80	95	95	95	60
45	62	29	50	10	100	80	65	95	70	60	30
46	63	22	65	20	90	80	70	50	80	70	40
47	59	16	70	40	70	70	30	50	65	80	60
48	52	16	75	20	65	55	40	55	45	60	50
49	72	25	80	65	100	55	20	90	95	85	60
50	72	12	70	45	90	75	70	80	75	70	70

The colours shown in the table correspond to the colours in Fig. 2.

Supplementary Table 2. Summary of inter-rater reliabilities in the assessment of tubular atrophy

	All Raters (n = 9)	Senior Raters (n = 4)	Junior Raters (n = 5)
ICC			
on all cases (tubular atrophy 0-100%)	0.57 (0.40–0.71)	0.62 (0.41–0.77)	0.47 (0.21–0.68)
on cases with moderate tubular atrophy (tubular atrophy 25-75%)	0.29 (0.15–0.47)	0.31 (0.10–0.54)	0.23 (0.06–0.44)
Pairwise Weighted Kappa (for Banff ct score)			
average on all cases (tubular atrophy 0-100%)	0.40 ± 0.18	0.47 ± 0.15	0.32 ± 0.19
average on cases with moderate tubular atrophy (tubular atrophy 25-75%)	0.22 ± 0.13	0.25 ± 0.16	0.15 ± 0.14
Pairwise Weighted Kappa (for Oxford T score surrogated by tubular atrophy)			
average on all cases (tubular atrophy 0-100%)	0.41 ± 0.18	0.48 ± 0.15	0.32 ± 0.19
average on cases with moderate tubular atrophy (tubular atrophy 25-75%)	0.22 ± 0.13	0.25 ± 0.16	0.15 ± 0.14

ICCs (intraclass correlation coefficients) showing in estimated value (95% confidence interval). Kappa statistics showing in mean ± standard deviation.

Supplementary Table 3. Inferred misclassification rates of Banff ci scores in reported large cohorts

				True Score					Misclassification	
				ci0	ci1	ci2	ci3		Number	%
2013 De Vusser et al. [1]				407	99	26	10			
(542)		*Inferred Score*	*ci0*	**256**	17	0	0	Downgrade	25	4.6%
			ci1	150	**67**	7	0	Upgrade	170	31.4%
			ci2	1	13	**14**	1	Total	195	36.0%
			ci3	0	1	5	**8**			
2013 Naesens et al. [2]				607	198	86	23			
(963)		*Inferred Score*	*ci0*	**382**	35	0	0	Downgrade	63	6.5%
			ci1	224	**134**	24	1	Upgrade	270	28.0%
			ci2	1	27	**46**	3	Total	333	34.6%
			ci3	0	3	15	**19**			

Numbers in parentheses are the total number of patients in publications. True ci scores reported in publications are underlined. Inferred scores in real-world practice are shown in italics. Case numbers in bold are correct classifications. The estimated misclassified case numbers and rates are shown on the right-hand side.

1. De Vusser K, *et al.* The predictive value of kidney allograft baseline biopsies for long-term graft survival. *J Am Soc Nephrol.* **23**, 1913-1923 (2013).
2. Naesens, M. *et al.* Chronic histological damage in early indication biopsies is an independent risk factor for late renal allograft failure. *Am J Transplant.* **13,** 86-99 (2013).

Supplementary Fig. 1 Curve charts showing the probability of making acceptable assessments with stricter (x ± 5) and looser (x ± 15) criteria

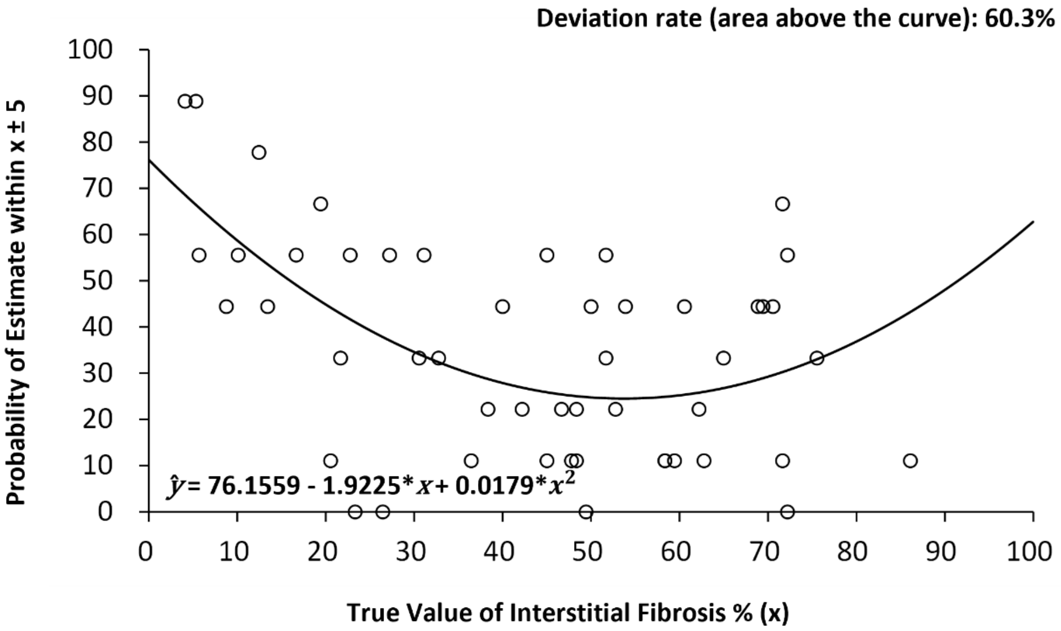

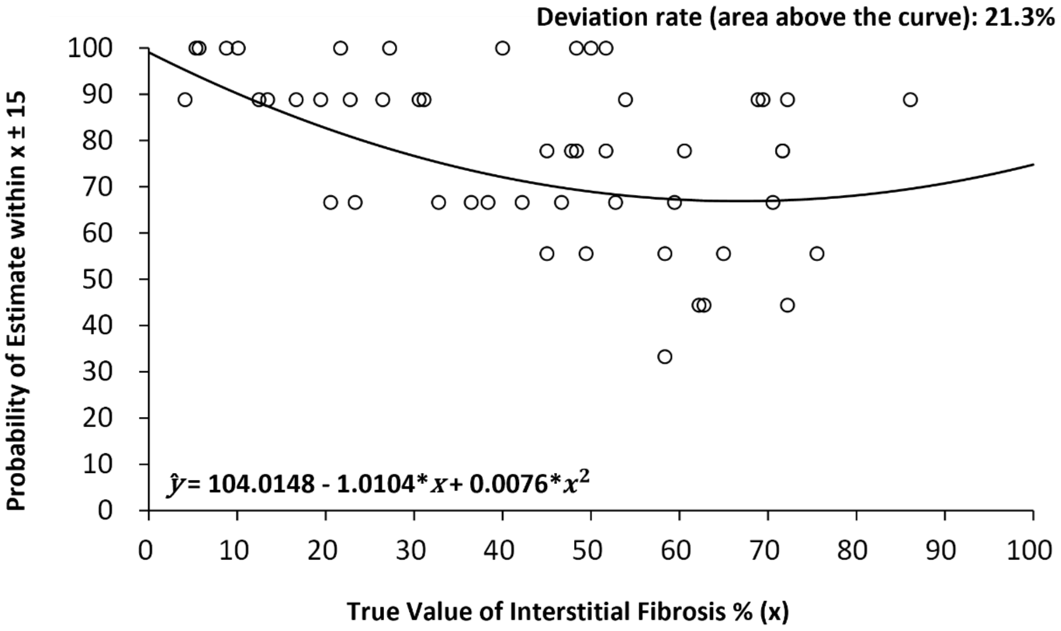

Supplement: Supplementary file 1 — Supplementary Information. [file 41598_2023_40221_MOESM1_ESM.pdf]
